# C-Fiber Loss as a Possible Cause of Neuropathic Pain in Schwannomatosis

**DOI:** 10.3390/ijms21103569

**Published:** 2020-05-18

**Authors:** Said C. Farschtschi, Tina Mainka, Markus Glatzel, Anna-Lena Hannekum, Michael Hauck, Mathias Gelderblom, Christian Hagel, Reinhard E. Friedrich, Martin U. Schuhmann, Alexander Schulz, Helen Morrison, Hildegard Kehrer-Sawatzki, Jan Luhmann, Christian Gerloff, Martin Bendszus, Philipp Bäumer, Victor-Felix Mautner

**Affiliations:** 1 Department of Neurology, University Medical Center Hamburg-Eppendorf, 20246 Hamburg, Germany; anna.hannekum@gmail.com (A.-L.H.); hauck@uke.de (M.H.); mgelderblom@uke.de (M.G.); janluhmann@gmx.net (J.L.); gerloff@uke.de (C.G.); v.mautner@uke.de (V.-F.M.); 2 Department of Neurology, Charité University Medicine, 10117 Berlin, Germany; tina.mainka@charite.de; 3 Berlin Institute of Health, 10178 Berlin, Germany; 4 Department of Neuropathology, University Medical Center Hamburg-Eppendorf, 20246 Hamburg, Germany; m.glatzel@uke.de (M.G.); hagel@uke.de (C.H.); 5 Department of Neurophysiology, University Medical Center Hamburg-Eppendorf, 20246 Hamburg, Germany; 6 Department of Maxillofacial Surgery, University Medical Center Hamburg-Eppendorf, 20246 Hamburg, Germany; r.friedrich@uke.de; 7 Department of Neurosurgery, University Medical Center Tübingen, 72076 Tübingen, Germany; martin.schuhmann@med.uni-tuebingen.de; 8 Leibniz Institute on Aging, Fritz Lipmann Institute, 07745 Jena, Germany; alexander.schulz@genetik-erfurt.de (A.S.); helen.morrison@leibniz-fli.de (H.M.); 9 MVZ Human Genetics, 99084 Erfurt, Germany; 10 Institute of Human Genetics, University of Ulm, 89081 Ulm, Germany; hildegard.kehrer-sawatzki@uni-ulm.de; 11 Department of Neuroradiology, University Medical Center Heidelberg, 69120 Heidelberg, Germany; martin.bendszus@med.uni-heidelberg.de (M.B.); p.baeumer@dialog-aoe.de (P.B.); 12 Department of Radiology, German Cancer Research Center, 69120 Heidelberg, Germany

**Keywords:** Schwannomatosis, small-fiber neuropathy, pain, MR-neurography, fascicular microlesions

## Abstract

Schwannomatosis is the third form of neurofibromatosis and characterized by the occurrence of multiple schwannomas. The most prominent symptom is chronic pain. We aimed to test whether pain in schwannomatosis might be caused by small-fiber neuropathy. Twenty patients with schwannomatosis underwent neurological examination and nerve conduction studies. Levels of pain perception as well as anxiety and depression were assessed by established questionnaires. Quantitative sensory testing (QST) and laser-evoked potentials (LEP) were performed on patients and controls. Whole-body magnetic resonance imaging (wbMRI) and magnetic resonance neurography (MRN) were performed to quantify tumors and fascicular nerve lesions; skin biopsies were performed to determine intra-epidermal nerve fiber density (IENFD). All patients suffered from chronic pain without further neurological deficits. The questionnaires indicated neuropathic symptoms with significant impact on quality of life. Peripheral nerve tumors were detected in all patients by wbMRI. MRN showed additional multiple fascicular nerve lesions in 16/18 patients. LEP showed significant faster latencies compared to normal controls. Finally, IENFD was significantly reduced in 13/14 patients. Our study therefore indicates the presence of small-fiber neuropathy, predominantly of unmyelinated C-fibers. Fascicular nerve lesions are characteristic disease features that are associated with faster LEP latencies and decreased IENFD. Together these methods may facilitate differential diagnosis of schwannomatosis.

## 1. Introduction

Schwannomatosis is a newly identified hereditary tumor predisposition disorder [1] with an estimated incidence of about one in 60,000 individuals [2]. Recognized as a neurofibromatosis-spectrum disorder, it is characterized by the occurrence of peripheral nerve schwannomas and meningiomas with or without spinal and cranial nerve involvement [3]. 

The clinical diagnostic criteria for schwannomatosis have changed over time since its first description as separate disease entity besides neurofibromatosis type 2 (NF2) [1,4,5]. In contrast to neurofibromatosis type 1 (NF1) and NF2, schwannomatosis is characterized by genetic heterogeneity. Thus far, disease-causing germline mutations have been identified in the genes *SMARCB1* and *LZTR1* [6,7,8,9]. However, in approximately 60% of sporadic and 14% of familial schwannomatosis cases, the disease-causing genes have not been identified yet [10]. 

The most common symptom of schwannomatosis is pain, reported by approximately 70% of patients; either as local, multifocal, or diffuse pain. While most patients remain asymptomatic during childhood and adolescence, adulthood invokes severe and poorly localized pain symptoms, sometimes in combination with pain attacks. Evidently, in the majority of schwannomatosis patients, pain is not associated with macroscopically detectable tumors [3]. The exact pathogenic cause of schwannomatosis-associated pain is, therefore, currently unknown. In the present study, we analyzed 20 schwannomatosis patients by means of neurophysiological, histological, and imaging methods to elucidate the morphological and neurophysiological correlates of schwannomatosis-associated pain. Additionally, we used questionnaires for further phenotypic characterization of the schwannomatosis-related pain syndrome and to identify comorbid psychological conditions, such as depression and anxiety. 

## 2. Results

### 2.1. Demographics and Course of Disease

All 20 patients enrolled in this study fulfilled the current clinical diagnostic criteria for schwannomatosis [11], three of which exhibited segmental schwannomatosis. The median age at diagnosis was 43 years (range: 26–70 years); the median age of symptom onset was 38 years (range: 16–61 years). Three of the 20 patients were familial cases whereas 17 patients were sporadic. Mutation analysis using blood-derived DNA did not indicate germline NF2 or SMARCB1 mutations in any of the 20 patients. LZTR1 germline mutations were identified in five of 20 patients. Demographics, clinical symptoms, and genetic analysis are summarized in Table 1.

Surgical schwannoma resection had been performed in 19 patients (ranging from one to 16 surgeries per patient), which includes peripheral nerve surgery in 14 patients, thoracic surgery in 11 patients, and cranial surgery in four patients. While pain relief and/or improvement of neurological function was reported by 16 of 18 patients following surgery, six of 18 patients reported new symptoms after surgery, predominantly pain and/or sensory loss or tingling. 

All patients had used analgesic or co-analgesic drugs in the past for the purpose of pain relief. NSAIDs and/or opioids were used by five of the 20 patients on a regular basis, whereas seven of the 20 patients used them on demand. One patient took gabapentin or pregabalin on a regular basis, one patient took these drugs on demand, and three had stopped the use of these drugs because of side effects or insufficiency of the therapy. Neuroleptics and antidepressants were used by one patient only.

### 2.2. Pain- and Personality Questionnaires

We firstly intended to characterize the pain symptoms experienced by schwannomatosis patients by established pain and personality questionnaires. In the painDETECT, six of 20 patients scored in the upper range (19–38 points), which is indicative of a neuropathic pain syndrome. Seven patients had low scores (0–12 points) and, thus, showed no symptoms typically associated with neuropathic pain. The remaining seven patients reached scores in the mid-range (13–18 points), allowing no precise classification. 

Schwannomatosis patients showed lower values in all scales of the SF-36 health survey compared to normative data and to the control group indicating a relevant impact on quality of life. Assessment of the HADS and SCL-90-R questionnaires showed a significantly higher vulnerability for anxiety and depression in the schwannomatosis cohort compared to controls (HADS: depression t(38) = −3.063, *p* = 0.004, anxiety U = 126.5, *p* = 0.046; SCL-90-R depression t(37) = −3.255, *p* = 0.002, anxiety t(37) = −2.297, *p* = 0.027). The FPI questionnaire did not detect any association with specific personality traits. Conclusively, the pain syndrome observed in patients with schwannomatosis has neuropathic features. Furthermore, anxiety and depression were over-represented in schwannomatosis patients leading to reduced quality of life in patients affected by schwannomatosis. 

### 2.3. Neurophysiological Measurements

The painDETECT questionnaire results indicated a neuropathic condition in a subset of schwannomatosis patients. In order to determine the functional integrity of peripheral nerves in schwannomatosis patients we performed standard electrophysiological measurements. In fact, 16 of 20 patients showed normal electrophysiological parameters. Only in four patients could signs of focal or multifocal sensomotoric axonal neuropathy could be detected. In three patients (#5, #10, #15) only one or two nerves were affected and associated with previous surgery in that area or with compressive schwannoma formation. Only one patient (#19) exhibited signs of more generalized neuropathy. 

### 2.4. Quantitative Sensory Testing 

**Figure 1:**
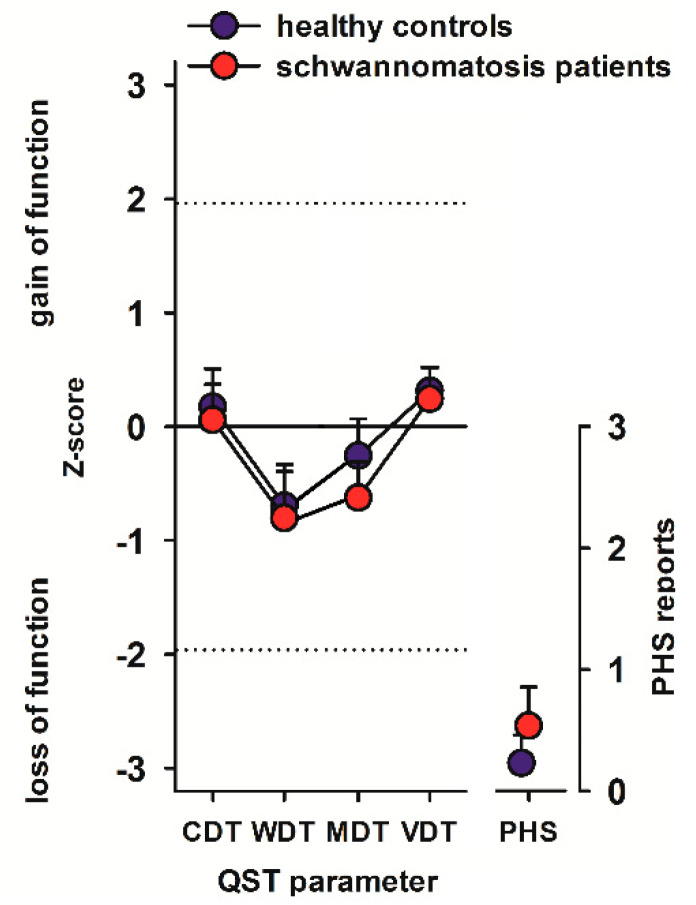
QST profile. CDT: cold detection threshold, WDT: warm detection threshold, MDT: mechanical detection threshold, VDT: vibration detection threshold, PHS: paradoxical heat sensations. Z-scores between −1.96 and +1.96 represent the normal range of healthy subjects. All values are normalized for age, gender and testing site on a Z-scale. Z-scores > 1.96 indicate a gain of sensory function; i.e., the subject detects the stimulus at a lesser stimulus intensity than healthy controls in case of detection thresholds (hyperaesthesia). By contrast, Z-scores < −1.96 indicate a loss of sensory function; i.e., the subject detects the stimulus at a greater stimulus intensity than healthy controls in case of detection thresholds (hypoaesthesia).

Standard electrophysiological measurements found pathological alterations in only a small subset of schwannomatosis patients. Quantitative sensory testing (QST) was applied as standardized noninvasive method to examine the function of thickly-myelinated Aβ, thinly- and unmyelinated Aδ- and C-fibers as well as their corresponding central pathways. Strikingly, QST did not detect any significant differences between schwannomatosis patients and healthy controls with regard to cold and warm temperature detection thresholds (*t*(76) = 0.496, *p* = 0.621 and *t*(76) = 0.384, *p* = 0.702, respectively) as parameters for small fiber function. No differences were observed in the occurrence of pathological paradoxical heat sensations (χ^2^(1) = 2.646, *p* = 0.104). Mechanical (*t*(76) = 1.585, *p* = 0.117) or vibration detection thresholds (*U* = 718, *p* = 0.671) as parameters for large fiber function also showed no differences between patients and controls (Figure 1). 

The number of individuals with signs of small, large, or mixed fiber neuropathy was not higher in the group of patients with schwannomatosis than in the group of healthy controls (χ^2^(3) = 3.227, *p* = 0.358).

### 2.5. Magnetic Resonance Imaging and Magnetic Resonance Neurography

**Figure 2:**
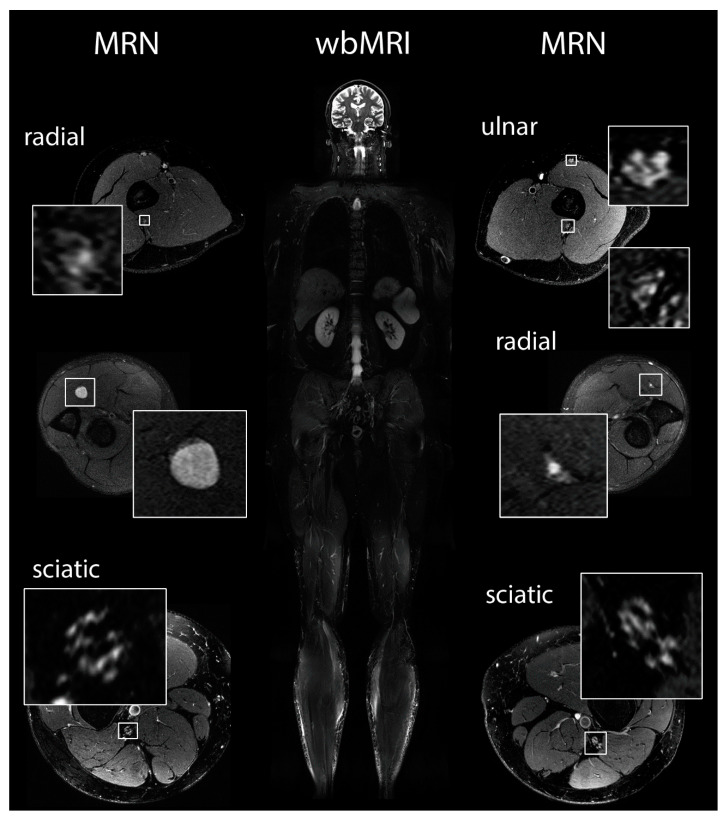
Scheme of imaging strategy. wbMRI and MRN protocols were performed for comprehensive imaging evaluation. wbMRI (middle column) was used for detection of large tumors. MRN (left and right columns) was used for detection of smaller nerve lesions. In this patient, fascicular nerve lesions are present in all nerves, while a larger tumor is shown in the right radial and an intermediate-sized nodule in the left radial nerve.

We next aimed at determining how chronic pain experienced by schwannomatosis patients correlates with the overall tumor burden. Therefore, whole-body MRI (wbMRI) investigations were performed in all 20 patients and additional MR neurography (MRN) was conducted in 18 patients (Figure 2). Seven patients had small intradural schwannomas of the cauda equina but compressive larger intradural lesions were not observed. Six patients exhibited subcutaneous schwannomas and seven patients had intramuscular schwannomas. Importantly, there was no correlation between tumor load or location and subjective pain perception. 

**Figure 3:**
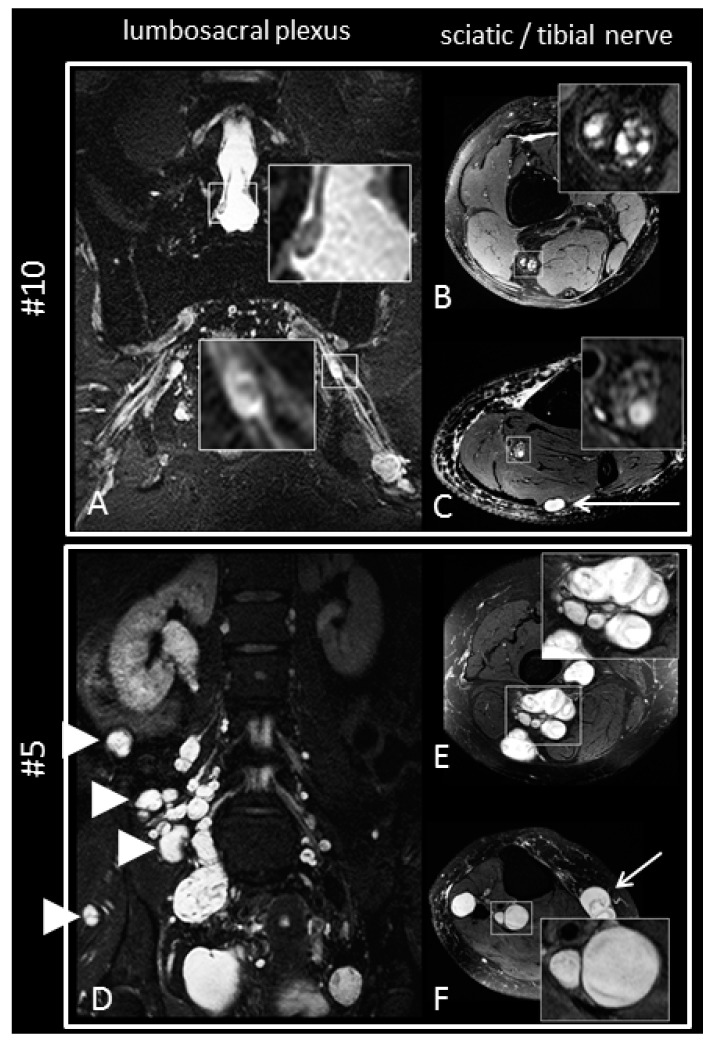
Exemplary cases of severe schwannomatosis imaging findings. The upper panel shows representative images of patient #10 with a high extent of nerve abnormalities (overall MRN lesion burden score 3). Schwannomas of the cauda equina (**A**, upper inset) and the sciatic nerve trunk (**A**, lower inset) are shown in magnification. Sciatic and tibial nerves have enlargement of individual fascicles (**B**,**C**). Lower panel shows patient with ubiquitous severe enlargement of all nerve structures (overall MRN score 4). All plexus elements as well as small intramuscular nerve branches are enlarged (**D**). Sciatic and tibial nerve (**E**,**F**, insets) are likewise transformed into masses affecting all nerve fascicles. Smaller nerves such as the saphenous nerve and sural nerve (**C**,**F**, arrows) as well as smaller muscular branches are clearly visible as schwannoma masses.

MRN indicated abnormalities in larger peripheral nerves (lumbosacral plexus, sciatic nerve, tibial, nerve, peroneal nerve, sural nerve) in 16 of 18 patients. These abnormalities ranged from small T2-weighted lesions of individual fascicles without caliber increase to large compressive schwannoma formations. Larger intraneural macrolesions or intermediate-sized nerve nodules were detected in MRN scans of 10 of 18 patients (Figure 3). In six patients, only fascicular nerve microlesions of less than 5 mm in diameter were observed. Two patients had no detectable nerve lesions on MRN scans at all. 

### 2.6. Laser Evoked Potentials 

In order to specifically test the integrity of nociceptive pathways in schwannomatosis patients, we performed laser-evoked potentials (LEP), which can be used to determine the presence of small-fiber neuropathy as it specifically measures the function of A-delta and C-fibers. Regarding LEP amplitudes, no differences were detected in the patient group compared to matched controls (N2: F(1,32) = 0.224, *p* = 0.640; P2: F(1,31) = 0.110, *p* = 0.742; N2P2: F(1,31) = 0.007, *p* = 0.933). Latencies tended to be faster in the patients group, but did not reach significance in the group comparison (N2: F(1,32) = 1.393, *p* = 0.247; P2: F(1,31) = 1.313, *p* = 0.261). In the individual plotting, 10 of 18 patients showed N2 latencies below the respective 95% confidence interval of the control group, whereas only two2 lay above. The remaining six patients showed latencies within the 95% confidence interval (Table 1). In the light of the small sample size this gives support that LEP latencies might be shorter in schwannomatosis patients. Taken together, faster A-delta nerve conduction as well as stable amplitudes of A-delta LEP signatures may indicate a predominant loss of C-fiber function as the cause for the development of neuropathic pain in schwannomatosis.

### 2.7. Histopathology of Intraepidermal Nerve Fiber Density

**Figure 4:**
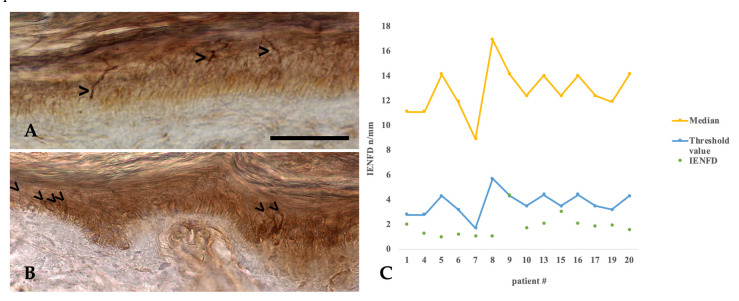
Intraepidermal nerve fiber density (IENFD). (**A**) Immunhistochemical staining for a 31 years old patient with schwannomatosis. Analysis of intraepidermal nerve fiber density (IENFD). The arrowheads mark three intraepidermal nerve fibers (mean fiber density 1.5/mm in the biopsy), scale bar 50 μm. (**B**) Immunhistochemical staining from a 37-year-old healthy volunteer. The arrowheads mark six intraepidermal nerve fibers (mean fiber density 6.7/mm in the biopsy), the scale is the same as in A. (**C**) IENFD (mean) with age-adjusted reference values (threshold is the 5% normative of the age and sex dependent reference group and mean of the reference group).

To further corroborate our findings that schwannomatosis-related pain might be caused by a loss of C-fiber function, we performed skin biopsies in 14 of 20 patients in order to determine the intraepidermal nerve fiber density (IENFD) as an alternative proxy for the presence of a small-fiber neuropathy in schwannomatosis patients. Strikingly, 13 of 14 patients who underwent skin biopsy exhibited a significant reduction of intraepidermal nerve fiber density (1.85 ± 0.92/mm) in comparison to age-adjusted reference-values (1.7–5.7/mm) [12] with a mean deviation from the age-dependent reference value of 1.84 ± 1.24/mm (Figure 4). Please note that the skin biopsy results presented here are a subset of data published in 2020 [13]. Interestingly, the observed reduction in intraepidermal nerve fiber density (IENFD) was neither associated with lesion burden as assessed by MR neurography (MRN), nor did it show any significant correlation with laser-evoked potentials (LEP) parameters.

## 3. Subjects, Materials and Methods 

### 3.1. Patients

In total, 20 patients (mean age 55.4 *±* 10.9 (SD) years; male:female = 1:1) were consecutively included in the study at the Neurofibromatosis outpatient clinic of the University Medical Center Hamburg-Eppendorf. Inclusion criteria were the fulfillment of the clinical diagnostic criteria for schwannomatosis [11] and absence of any other disease with potential impact upon central or peripheral nervous system function, such as chronic infections, autoimmune diseases, malignancies, diabetes, or alcoholism. Detailed neurological examination was performed by a physician (VFM) with 30 years of experience in the diagnosis and treatment of neurofibromatosis and schwannomatosis. In order to perform genetic testing, genomic DNA of the patients was isolated from peripheral blood lymphocytes and used as a template for the PCR amplification of *NF2*, *SMARCB1,* and *LZTR1* exons. Sanger sequencing of the PCR products was performed using the BigDye Terminator Cycle Sequencing Kit (ABI, Life Technologies, Foster City, CA, USA). Rearrangments involving the NF2 gene were investigated by MLPA (SALSA MLPA P044 NF2 probemix, MRC Holland, Amsterdam, The Netherlands).

Control groups were method-specific cohorts of healthy controls (QST, LEP, MRN, questionnaires) and established reference values (questionnaires, neurophysiological measurements, IENFD). For MRN and IENFD, measurements and analyses were conducted in a blinded manner. For the QST, LEP, and neurophysiological examination blinding the investigators was not possible, however, data analysis was carried out in a blinded fashion.

### 3.2. Pain- and Personality-Questionnaires

The Hospital Depression and Anxiety Score (HADS) was used to screen for anxiety and depression [14]. Pain perception was assessed by the PainDETECT questionnaire, which provides high sensitivity, specificity, and positive predictive value to detect neuropathic pain [15]. In order to examine stable traits of patients’ personality the multidimensional “Freiburger Persönlichkeitsinventar” (FPI) was used [16]. By means of the Symptom Checklist-90-R (SCL-90-R) [17], the patients’ subjective strain through physical and psychological disorders were measured. The German Version of the SF-36 health survey was utilized to assess health-related quality of life [18]. A group of 20 age and sex-matched healthy controls (mean age 56 *±* 11.2 (SD) years; male:female = 1:1) served as a control group. 

Normal distribution was investigated using the Kolmogorov–Smirnov test. Then, questionnaire data was compared between groups using either unpaired *t*-tests or Mann–Whitney-U test, as appropriate. *p*-values < 0.05 were considered statistically significant. 

### 3.3. Neurophysiological Measurements

Conventional electrodiagnostic testing (nerve conduction velocity and compound muscle action potential amplitude) of the right peroneal, left tibial, and right ulnar and/or median nerve was performed in the neurophysiology laboratory of the Hamburg-Eppendorf University Hospital using a Nihon Kohden Neuropack X1 (Rosbach v.d.H., Germany).

### 3.4. Quantitative Sensory Testing (QST)

QST is a standardized noninvasive psychophysical method, which examines the function of thickly-myelinated Aβ, thinly- and unmyelinated Aδ-, and C-fibers as well as their corresponding central pathways by the application of thermal and mechanical stimuli on the skin. QST was performed by a certified examiner (ALH) after a pre-defined protocol by the German Network on Neuropathic Pain (DFNS) [19] in all 20 patients. QST comprised seven different tests measuring the following 13 parameters: cold and warm detection thresholds (CDT, WDT), cold and heat pain thresholds (CPT, HPT), paradoxical heat sensations (PHS) during the thermal sensory limen (TSL) procedure, mechanical detection threshold (MDT), mechanical pain threshold (MPT), stimulus-response-functions for pinprick sensitivity (MPS) and dynamic mechanical allodynia (DMA), pain summation to repetitive pinprick stimuli (wind-up ratio, WUR), vibration detection threshold (VDT), and pressure pain threshold (PPT).

A group of 20 age- and sex-matched healthy controls (mean age 54.8 *±* 11.2 (SD) years; male:female = 1:1) served as control group. QST was assessed on the dorsum of both hands and feet except in one patient (and its matched control) in whom only both hands were assessed. Areas whose innervation could have been compromised by previous surgeries (e.g., scars) were avoided. 

QST statistical analysis was performed using “eQUISTA“ (Casquar GmbH, Bochum, Germany), which calculates a sensory profile by transformation of the absolute measured values into relative data by calculation of z-scores according to the following formula based on the published data of healthy controls: z-value = (value_patient_ – mean_controls_) / SD_controls_ [20]. CDT, WDT, and PHS as parameters for function of small nerve fibers and MDT and VDT as parameters for function of large nerve fibers were statistically investigated. Normal distribution was ascertained via Kolmogorov–Smirnov test. Then, either unpaired *t*-tests or the non-parametric Mann–Whitney U-test were used to compare the pooled z-values of hands and feet between patients and controls, as appropriate. Pathological PHS and the number of patients with small, large and mixed fiber neuropathy (according to QST) as categorical variables were compared between groups using the Chi-square test. *p*-values < 0.05 were regarded as statistically significant.

### 3.5. Laser Evoked Potentials (LEP)

LEP were recorded in 18 patients and matched controls. Two patients were refused to undergo LEP measurements. We delivered brief infrared laser stimuli of 1 ms duration and a beam diameter of 5 mm to the dorsum of both feet and hands using a thulium YAG laser (wavelength 2 μm, StarMedTec, Starnberg, Germany). Individual pain thresholds were determined using three series of increasing and decreasing stimuli. Beginning at 160 mJ, we used a step-size of 20 mJ. Pain was defined as a feeling of a light pin-prick or burning. LEP are capable to investigate nociceptive pathways, especially A-delta fibers.

During the experiment, subjects were comfortably seated in an electrically shielded and sound-attenuated recording chamber with eyes closed. The session consisted of eight blocks and started at the left hand, followed by the right hand, right foot, and left foot. Repetitions of all sites were then performed in the reverse order to minimize confounding of site with habituation effects over time. Each block comprised 30 laser-stimuli with a two-fold pain threshold intensity. The inter-stimulus interval varied between six or seven seconds. Two seconds after the laser stimulus, an acoustic event (2000 Hz tone) prompted a verbal response, scaling pain intensity and ensuring patients attention. EEG was recorded with nose-reference using 64 active channels (EASY CAP) and BrainVision Recorder-software (Brain Products GmbH, Gilching, Germany) through two BrainAmp MRplus 32 channel amplifiers with a sampling frequency of 1000 Hz and a band pass filter between 0.1–250 Hz. The electrode impedance was kept below 15 kΩ. The data were analyzed offline using Fieldtrip software (Donders Centre for Cognitive Neuroimaging, AH Nijmegen, The Netherlands, www.ru.nl/fcdonders/fieldtrip). For detailed artifact rejection see Hauck and colleagues [21].

Mixed analysis of variance was used to compare LEP amplitudes and LEP latencies between patients and controls with amplitude (or latency) as 4-level dependent variable and group as independent variable. *p*-values < 0.05 were regarded as statistically significant.

### 3.6. Magnetic Resonance Imaging and Neurography

Vestibular schwannomas—the hallmark tumor entity of NF2—were excluded by contrast-enhanced cranial MRI. Patients underwent whole-body magnetic resonance imaging (wbMRI) at 3.0 Tesla according to previously described protocols [22]. Peripheral schwannomas detectable on wbMRI were counted to estimate total tumor burden. Additional high-resolution microstructural MR neurography was performed in 18 of 20 patients at 3.0 Tesla (Magnetom Verio, Siemens, Erlangen, Germany) for the lumbosacral plexus and the right lower extremity. Additional extremities were optionally examined based on patient’s compliance and ability to lie still for prolonged examination time. Doing so the contralateral leg was also examined in another eight patients and the upper extremities were additionally examined in five of those patients. The sequence protocol was as follows:

Lumbosacral plexus: 3D-isotropic T2-weighted SPACE sequence with fat-saturation by inversion-recovery and TR/TE/TI 3800/267/210, voxel size 0.95 × 0.95 × 1.0 mm, FoV 250 × 245 mm, acquisition time 8:32 min.

Extremities were examined by multiple stacks of 2D T2-weighted turbo-spin-echo sequences (for the right leg generally thigh, knee, calf level) with the following parameters: T2 TSE TR/TE 7552/52, in-plane resolution 0.273 × 0.273 mm^2^, FoV 140 mm, slice thickness 3.0 mm, frequency-selective fat-saturation, acquisition time 7:07 min.

Dedicated surface array coils for plexus imaging and circularly-polarized extremity transmit-receive coils for extremities were used. MR neurography (MRN) scans were evaluated blinded to the patients’ data by two radiologists (PB, MB) with more than eight and 18 years of experience in MRN, respectively, to detect:

(1) Manifest peripheral nerve tumors of more than 5mm in diameter;
(2) Intermediate-sized nerve nodules, defined as neural caliber increase between 2 and 5 mm in diameter; and
(3) The presence of peripheral nerve microlesions with increased T2-weighted signal but with only minor or no fascicular caliber increase (< 2 mm in diameter as described previously [23]) (Figure 2).

This quantitative analysis was performed for the right lower extremity, which was examined in all patients except for one whose contralateral leg was examined due to a right hip prosthesis. Manifest peripheral nerve tumors and nerve nodules were counted individually unless they exceeded more than ten. In addition, an overall MRN score for lesion accumulation of peripheral nerves of the right leg was used with regard to the presence of tumors, intermediate lesions, and microlesions on a Likert scale of 0–4 (with 0 indicating no nerve abnormalities, 1 slight but distinct abnormalities, 2 disseminated nerve lesions, 3 high extent of nerve abnormalities with only few nerve segments without clear lesions, and 4 massive ubiquitous tumor burden). For MRN statistical analysis, Pearson correlation and unpaired t-test were used to investigate association of MRN lesion load with LEP parameters, IENFD, or tumor load in wbMRI.

### 3.7. Skin Biopsies and Intraepidermal Nerve Fiber Density

Skin biopsies were performed in 14 of 20 patients by an expert surgeon for plastic surgery (RF). Six patients refused to have skin biopsies. An 8 mm punch biopsy was taken 10 cm above the lateral malleolus of the distal leg according to the standardized protocol for diagnosis of small fiber neuropathies (European Federation of Neurological Societies/Peripheral Nerve Society Guideline) [24]. The tissue was directly transferred into Zamboni solution and cooled to 4 °C. Immunohistochemical staining and evaluation was performed at the Institute of Neuropathology, University Medical Center Hamburg-Eppendorf, by using a standardized protocol. In short, 50 µm thick frozen cross sections of the skin sample were cut on superfrost slides additionally coated with poly-L-lysine (Sigma-Aldrich, #P8920, Taufkirchen, Germany) and air dried for at least 24 hrs. Blocking of endogenous peroxidase was done with 3% perhydrol (Merck, #107209, Darmstadt, Germany) in methanol for 90 min. The tissue was rehydrated and pre-treated for antigen-retrieval in 10 mM citrate buffer pH 6 for 90 min at 80 °C. The samples were then incubated with polyclonal rabbit anti-PGP 9.5 (Dako #Z5116, Glostrup, Denmark, antibody dilution 1:200) over night at room temperature. Bound antibody was visualized with anti-rabbit secondary antibody (Histofine Max PO Multi, #414152 F, Nichirei Biosciences Inc., Tokyo, Japan) for three hours followed by addition of the chromogen Metal Enhanced DAB Substrate Kit (Thermo Scientific, #34065, Bremen, Germany) according to manufacturer’s instructions for 15 min. Counterstaining was performed with haemalum. Antibody labelling of dermal nerve fascicles served as internal positive control and was differentiated from weak unspecific stroke-like staining of collagen fibers and chromogen deposition in small tissue tears and clefts by the higher intensity of labelling, the stretch from subepidermal to intradermal layer and the characteristic curved shape of the fibers with sudden turns and bends (Figure 4A). For estimation of nerve fiber density the intraepidermal nerve fiber count per mm skin was evaluated in at least three cuts corresponding to a skin length of approx. 9 mm by two experienced neuropathologists (MG and CH) who were blinded to patient identity and diagnosis. The intraepidermal nerve fiber density (IENFD) was compared to age-dependent reference values [12]. The raw IEND data were plotted against age, separately for male and female patients and compared to the normative reference IEND. Some parts of this analysis have already been published in a comparative study with patients exhibiting NF2 [13]; that protocol focused on establishing a new diagnostic approach for differential diagnosis and is a follow-up protocol to this study.

### 3.8. Standard Protocol Approvals, Registrations, and Patient Consents

The study was approved by the ethical board of the Medical Association Hamburg (PV4421), Germany, and performed in accordance with the Declaration of Helsinki. Written informed consent was obtained from all study participants

### 3.9. Data Availability Statement

The datasets generated during and/or analyzed during the current study are available from the corresponding author on reasonable request.

## 4. Discussion

In this study we aimed at investigating the pain characteristics and peripheral nerve abnormalities in patients with schwannomatosis by clinical and genetic examination, psychological testing, detailed electrophysiological measures, wbMRI and MRN, as well as by analyzing the intraepidermal nerve fiber density. We hereby provide evidence that point toward a C-fiber neuropathy as the underlying cause for the pain experienced by most schwannomatosis patients.

According to the evaluation of the painDETECT questionnaire, the pain syndrome observed in patients with schwannomatosis has neuropathic features. Anxiety and depression were over-represented in schwannomatosis patients according to the HADS questionnaire and the SCL-90-R. Conclusively, the applied questionnaires indicated a significant pain-burden with a risk of comorbid depression in schwannomatosis. From the SF-36 we conclude that pain reduces the quality of life in patients with schwannomatosis considerably and that the development of effective pain treatment protocols are essential. According to the FPI, the pain perception cannot be referred to specific traits of personality.

By standard neurophysiological assessment, a true sensorimotor polyneuropathy was not detectable in our patients except in one. Remarkably, we did not detect signs of neuropathy by means of quantitative sensory testing (QST) either, even though the sensitivity of QST is considered high [25]. In the more common forms of neuropathies (e.g., diabetic polyneuropathy) sensory deficits are distally pronounced and thus can be detected by QST of the feet. However, in schwannomatosis neuropathy might have a patchier distribution, leading to negative results of QST in this specific testing area.

Importantly, the subjective location of pain symptoms and the extent of pain experienced by the schwannomatosis patients did not correlate with the location of tumors as analyzed by wbMRI. Instead, by using high-resolution MRN we detected generalized fascicular nerve lesions in most patients. These lesions are morphologically comparable to those found in patients with NF2 [21]. However, in contrast to the fascicular lesions observed in NF2, those identified in patients with schwannomatosis were not associated with objective neurological deficits such as limb weakness or sensory loss. We even found small fascicular nerve microlesions in clinically asymptomatic extremities that were not affected by tumors similar to lesions found in previously described patients exhibiting segmental schwannomatosis [26].

In performing laser evoked potentials (LEP), we identified that the N2/P2 latencies might be faster in the schwannomatosis patient group compared to matched controls but without reaching significance due to the small sample size. This may contradict the conventional concept of small fiber neuropathy, but may be explained by an exclusive impairment of C-fibers without major affection of the A-delta fibers [27]. Laser stimulation of the skin mainly activates nociceptive A-delta and C-fibers. Since C-fibers jitter, they cannot be detected using N2/P2 components of the pain evoked potential [28]. Therefore, the N2/P2 component is mainly a correlate of A-delta fiber function. Furthermore, A-delta fiber and C-fiber components exhibit a competitive signature in electrophysiological measurements [28,29]. This means, if A-delta fiber input decreases, C-fibers can be unmasked, and vice versa. Another possible explanation may be due to changes in neuronal plasticity. Patients suffering from C-fiber loss adapt by enhanced A-delta fiber input processing to evaluate pain, since the measured laser N2/P2 is generated in the cingulate gyrus [30] and contains cognitive pain evaluation.

Our assumption that schwannomatosis-related pain might be caused by a loss of C-fiber function and subsequent small-fiber neuropathy is substantiated by the results of intraepidermal nerve fiber density measurements, which are currently considered the gold standard for the diagnosis of small-fiber neuropathies [12,24]. In our study, all schwannomatosis patients showed considerable reduction of the intraepidermal nerve fiber density, indicative of chronic small-fiber neuropathy as cause for the neuropathic pain experienced by schwannomatosis patients. Moreover, we found strong evidence that this reduction of intraepidermal nerve fiber density is pathognomonic for schwannomatosis in the spectrum of similar disorders especially in contrast to neurofibromatosis type 2 [13]. In analogy to other small-fiber entities [31,32], the combination of clinical pain pattern and the observed LEP abnormalities strongly suggests the presence of small-fiber neuropathy as an intrinsic disease feature of schwannomatosis.

Differential diagnosis of schwannomatosis can be difficult in patients, especially if they are oligosymptomatic and testing for *SMARCB1* and *LZTR1* germline mutations is negative. Additionally, schwannomas are frequent sporadic tumors [33] and may be associated with other syndromes as well [34,35,36,37,38].

In comparing NF1, NF2, and schwannomatosis with regard to the respective diagnostic features and symptoms, it is reasonable to hypothesize that C-fiber loss is indeed the cause for pain experienced by schwannomatosis patients.

Of all three neurofibromatous entities, schwannomatosis is the only one presenting primarily with pain [39]. However, in the majority of patients the macroscopic tumor growth regarding potential peripheral nerve compression, as measured by wbMRI, is at least comparable in all three NF subtypes, or even larger in NF1. Nerve microlesions, as detected by magnetic resonance neurography (MRN), are present in both NF2 and schwannomatosis patients, but not NF1. Strikingly, while many NF2 patients may suffer from sensorymotor neuropathy, individuals affected by schwannomatosis do not.

Consequently, both the macroscopic tumor burden as well as nerve microlesions are unlikely to be responsible for pain symptoms seen in schwannomatosis patients. The decisive diagnostic feature, which separates schwannomatosis clearly from NF1 and NF2, is the marked reduction of IENFD.

Neuropathic pain is defined as pain caused by a lesion or disease of the somatosensory nervous system. Here, we demonstrated this premise for neuropathic pain with the marked loss of epidermal nerve fibers in schwannomatosis patients. The hypothesis that peripheral nerve tumors lead to neuropathic pain has been raised before [40]. There are several possible mechanisms that might be responsible for the development of neuropathic pain: First, tumor-related injury of Schwann cells might lead to increased production of inflammatory cytokines that are known to promote neuropathic pain [41,42]. Second, tumorous Schwann cells might secrete less neurotrophic factors [43], impairing the ability to guide sprouting axons. This might prevent the formation of functional connections, leading to abnormal signaling [44].

Further research on cytokine expression and nerve growth in schwannomas might shed light on this interesting, yet still disregarded topic.

The findings presented in our study strongly suggest that reduced intraepidermal small-fiber density and the presence of multiple fascicular nerve lesions are important diagnostic signs indicative of schwannomatosis. Hence, skin biopsies for the analysis of intraepidermal nerve fiber density as well as MR neurography for detection of fascicular nerve lesions might represent powerful diagnostic tools to strengthen the diagnosis of schwannomatosis.

## 5. Conclusions

In our cohort of 20 schwannomatosis patients we found no significant neurological deficits directly attributable to schwannomatosis. However, the extensive work-up revealed evidence for the existence of a chronic neuropathic pain syndrome in all patients. Larger tumors and disseminated intraneural fascicular nerve lesions shown by wbMRI and MRN, reduced intraepidermal nerve fiber density in the skin biopsy, and possible C-fiber dysfunction in LEP demonstrate affection of nerve fibers, yielding the base for the diagnosis of neuropathic pain. Our study, therefore, indicates that a loss of C-fibers might play a role in the pathogenesis of pain in patients with schwannomatosis. High-resolution microstructural MR neurography and IENFD are potential surrogates to allow differential diagnosis of schwannomatosis in uncertain cases.

## Figures and Tables

**Table 1:** Baseline characteristics of all included patients. Sex, age at examination, family history, germline mutations in *LZTR1*, *SMARCB1*, or *NF2* genes: Sanger sequencing using blood-derived DNA; rearrangements involving the *NF2* gene were not detected by MLPA (* if available tumor tissue was investigated for *NF2* mutations: patient #20 has two different *NF2* mutations in two schwannomas), symptoms: bold: first symptom, other: non-neurological symptoms, location of tumors, number of schwannomas detected in wbMRI, microlesions (MRN)/MRN right leg overall score: number of microlesions on hr-MRI (MRN)/mi-crolesions (0: none; 1: low lesion load; 2: intermediate lesion load; 3: high lesion load; 4: very high lesion load), neurophysiology (normal or patho-logical), QST: normal, small fiber neuropathy (SFN), mixed fiber neuropathy (MFN), or large fiber neuropathy (LFN), LEP: N2 latency (exemplatory of the left foot), semiquantitative description according to the 95% confidence interval of the normgroup: shorter (↓), normal (0) or longer (↑) latency, PainDETECT: 0: no evidence for neuropathic pain, +: inconclusive, ++: suggestive for neuropathic pain, IENFD: intraepidermal nerve fiber density (IENFD; n/mm) according to age and sex adjusted reference values (0: normal, ↓: on the 5% reference value; ↓↓: below the 5% normative); n/a: not available.

PAT_ID	SEX	AGE	FAMILY HISTORY	LZTR1	SMARC B1	NF2	SYMPTOMS	LOCATION Of TUMORS	NUMBER Of TUMORS (wb-MRI)	MRN: MICROLESIONS/RIGHT LEG OVERALL SCORE	NEUROPHYSIOLOGY	QST HANDS	QST FEET	LEP N2 LATENCY LEFT FOOT	PAINDETECT	IENFD
*1*	M	65	Yes	n.d.	n.d.	n.d.	**Pain**, local tumor growth	Peripheral	10	1/1	normal	MFN	normal	↑	++	↓↓
*2*	W	38	No	VUS c.1288C>T;His430Tyr	n.d.	n.d.	**Pain**, local tumor growth	Segmental	2	2/1	normal	normal	n/a	n/a	++	n/a
*3*	W	53	No	n.d.	n.d.	n.d.	**Pain**, local tumor growth	Peripheral	15	3/2	normal	normal	normal	n/a	+	n/a
*4*	M	66	No	n.d.	n.d.	n.d.	**Pain**, local tumor growth	Spinal and peripheral	32	3/2	normal	MFN	normal	0	0	↓↓
*5*	W	52	No	n.d.	n.d.	n.d.	**Pain**, local tumor growth	Spinal and peripheral	4	4/4	path.	normal	SFN	↓	+	↓↓
*6*	W	61	No	n.d.	n.d.	n.d.	**Other**, pain, local tumor growth	Segmental	5	1/0	normal	normal	normal	↓	++	↓↓
*7*	M	80	No	n.d.	n.d.	n.d.	**Other**, pain	Cerebral, peripheral, and spinal	5	2/1	normal	LFN	LFN	↓	+	↓↓
*8*	W	46	No	c.C2247A;p.Y749X	n.d.	n.d.	**Pain**	Segmental	5	2/1	normal	normal	normal	↓	0	↓↓
*9*	W	56	No	c.G1312T;p.E438X (Exon12)	n.d.	n.d.	**Pain**, local tumor growth	Spinal and peripheral	13	1/1	normal	normal	normal	↓	+	↓
*10*	M	56	No	n.d.	n.d.	n.d.	**Pain**, weakness/ sensory loss, local tumor growth	Spinal and peripheral	5	4/3	path.	SFN	normal	↑	0	↓↓
*11*	W	41	No	c.1480_1481insAGp.R494fs (Exon14)	n.d.	n.d.	**Pain**	Cerebral, peripheral, and spinal	8	n/a	normal	LFN	normal	0	+	n/a
*12*	M	64	No	n.p.	-	n.d.	**Pain**, local tumor growth	Peripheral	3	n/a	normal	SFN	LFN	0	0	n/a
*13*	M	42	Yes	c.978-985delCAGCTCCG (Pro326fs)	n.d.	n.d.	**Pain**, local tumor growth	Spinal and peripheral	61	1/0	normal	normal	SFN	↓	0	↓↓
*14*	W	46	No	n.d.	n.d.	n.d.	**Pain**	Spinal and peripheral	25	4/4	normal	MFN	LFN	↓	++	n/a
*15*	M	57	No	n.d.	n.d.	n.d.	**Weakness/sensory loss**, other, pain	Spinal and peripheral	3	1/1	path.	SFN	SFN	0	++	↓↓
*16*	M	47	No	n.d.	n.d.	n.d.	**Other**, pain	Peripheral	8	3/2	normal	normal	normal	↓	0	↓↓
*17*	M	56	No	n.d.	n.d.	n.d.	**Pain**	Spinal and peripheral	15	2/2	normal	normal	normal	↓	+	↓↓
*18*	M	73	Yes	n.d.	n.d.	n.d.	**Pain**, local tumor growth	Spinal and peripheral	33	3/2	normal	SFN	MFN	0	++	n/a
*19*	W	60	No	n.d.	n.d.	n.d.	**Pain**, other, local tumor growth	Spinal and peripheral	3	0/1	path.	normal	normal	↓	0	↓↓
*20*	W	49	No	n.d.	n.d.	n.d.*	**Other**, pain	Peripheral	3	0/0	normal	normal	normal	↓	+	↓↓

## Abbreviations

CDT	Cold detection thresholds
CPT	Cold pain thresholds
DFNS	Deutscher Forschungsverbund Neuropathischer Schmerz
DMA	Dynamic mechanical allodynia
FoV	Field of view
FPI	Freiburger Persönlichkeitsinventar
HADS	The Hospital Depression and Anxiety Score
HPT	Heat pain thresholds
IENFD	Intra-epidermal nerve fiber density
LEP	Laser evoked potentials
LZTR1	Leucine zipper like transcription regulator 1
MDT	Mechanical detection threshold
MLPA	Multiplex ligation-dependent probe amplification
MPS	Mechanical pain sensitivity (stimulus-response-functions for pinprick sensitivity)
MPT	Mechanical pain threshold
MRN	Magnetic resonance neurography
N2/P2	Brain responses to laser evoked potentials
NF1	Neurofibromatosis type 1
NF2	Neurofibromatosis type 2
NSAID	Nonsteroidal anti-inflammatory drugs
PGP 9.5	Protein gene product 9.5
PHS	Paradoxical heat sensations
PPT	Pressure pain threshold
QST	Quantitative sensory testing
SCL-90-R	SYMPTOM Checklist-90-R
SD	Standard deviation
SF-36	Short form health 36
SMARCB1	SWI/SNF-related matrix-associated actin-dependent regulator of chromatin subfamily B member 1
TE	Echo time
TI	Inversion time
TR	Repetition time
TSE	Turbo spin echo
TSL	Thermal sensory limen
VDT	Vibration detection threshold
wbMRI	Whole-body Magnetic Resonance Imaging
WDT	Warm detection thresholds
WUR	Wind-up ratio (pain summation to repetitive pinprick stimuli)
